# Extracellular vesicles in the development of organ‐specific metastasis

**DOI:** 10.1002/jev2.12125

**Published:** 2021-07-19

**Authors:** Fumihiko Urabe, Kalyani Patil, Grant A. Ramm, Takahiro Ochiya, Carolina Soekmadji

**Affiliations:** ^1^ Department of Urology The Jikei University School of Medicine Tokyo Japan; ^2^ Department of Molecular and Cellular Medicine Tokyo Medical University Tokyo Japan; ^3^ Department of Molecular Pathophysiology Translational Research Institute Academic Health System Hamad Medical Corporation Doha Qatar; ^4^ Department of Cell and Molecular Biology QIMR Berghofer Medical Research Institute Brisbane Queensland Australia; ^5^ School of Biomedical Sciences Faculty of Medicine University of Queensland Brisbane Queensland Australia

**Keywords:** Cancer, extracellular vesicles, intercellular communication, metastasis, organotropism

## Abstract

Distant organ metastasis, often termed as organotropic metastasis or metastatic organotropism, is a fundamental feature of malignant tumours and accounts for most cancer‐related mortalities. This process is orchestrated by many complex biological interactions and processes that are mediated by a combination of anatomical, genetic, pathophysiological and biochemical factors. Recently, extracellular vesicles (EVs) are increasingly being demonstrated as critical mediators of bi‐directional tumour‐host cell interactions, controlling organ‐specific infiltration, adaptation and colonization at the secondary site. EVs govern organotropic metastasis by modulating the pre‐metastatic microenvironment through upregulation of pro‐inflammatory gene expression and immunosuppressive cytokine secretion, induction of phenotype‐specific differentiation and recruitment of specific stromal cell types. This review discusses EV‐mediated metastatic organotropism in visceral (brain, lung, liver, and lymph node) and skeletal (bone) metastasis, and discusses how the pre‐metastatic education by EVs transforms the organ into a hospitable, tumour cell–friendly milieu that supports the growth of metastatic cells. Decoding the organ‐specific traits of EVs and their functions in organotropic metastasis is essential in accelerating the clinical application of EVs in cancer management.

## 1 INTRODUCTION

Metastasis to distant organs is a fundamental feature of malignant neoplasms and is its most lethal attribute that accounts for > 90% of mortality from cancer. The conceptual framework of tumour progression towards metastasis has been well‐described and simplified into a stepwise sequence of cell‐biological events, which are collectively termed the invasion‐metastasis cascade (Valastyan & Weinberg, 2011). Cumulative evidence has shown that each type of cancer exhibits striking disparities in the temporal course (relapse within a range of organs) of metastasis, kinetics (time to relapse/recur) of metastatic progression and the severity of metastasis in vital target organs (Nguyen et al., 2009). While genetics can determine the metastatic traits of each cancer type, these traits can be acquired by disseminated tumour cells (DTCs; tumour cells with a metastatic propensity residing in the permissive target tissues of distant organs) through organ‐selective evolution or adaptation of metastatic cell populations (Nguyen et al., 2009). A large body of evidence has underscored the significance of host tissue characteristics such as the heterogeneous populations of host cells at the secondary metastasis site (Labelle & Hynes, 2012). However, the exact nature of temporal and spatial interactions between the host and tumour cells, and the precise hierarchy and function of these interactions during metastasis remain incompletely understood. In recent years, the discovery and implication of a new mode of intercellular communication mediated by extracellular vesicles (EVs) at local and secondary metastasis sites has provided provocative insights into the tumour‐host cell interactions.

EVs are a heterogeneous family of vesicles that are generated from different subcellular compartments and released into the extracellular space (see more detailed reviews (Van Der Pol et al., 2012; Van Niel et al., 2018; Yáñez‐Mó et al., 2015)). EVs are secreted by all cells and found in abundance in bodily fluids including blood, bile, urine, breast milk, and synovial, ascites, lacrimal, seminal and bronchoalveolar lavage fluids (Soekmadji et al., 2020). EVs are released in response to various extrinsic and intrinsic cues such as low pH conditions, hypoxia, injury, irradiation, microenvironmental stress, cell activation (for example, platelet activation) and exposure to complement regulatory proteins (Harmati et al., 2019; Kucharzewska et al., 2013; Mittelbrunn et al., 2011). Exosomes (30‐150 nm), ectosomes or microvesicles (0.1–2 μm) and apoptotic bodies (1–5 μm) are the most studied EVs to date (Doyle & Wang, 2019; Simons & Raposo, 2009; Soekmadji et al., 2013; Yáñez‐Mó et al., 2015). These vesicles are characterized based on intracellular origin and their size. Exosomes are endosome‐derived vesicles that originate from intraluminal vesicles (ILVs) formed through the inward budding of the late endosomal membrane (multivesicular bodies (MVBs)). MVBs may either fuse with lysosomes resulting in the proteolytic degradation of ILVs, or traffic to and fuse with the plasma membrane to release the ILVs as exosomes in the extracellular milieu (Mathieu et al., 2019; Van Niel et al., 2018). Microvesicles are formed through the direct budding from the plasma membrane (Ratajczak & Ratajczak, 2020), whereas apoptotic bodies are generated by apoptotic cells via apoptotic cell disassembly (Atkin‐Smith & Poon, 2017). Recently, using asymmetric flow field‐flow fractionation (AF4), Zhang and colleagues discovered a population of non‐membranous secreted nanovesicles termed ‘exomeres’ (∼35 nm) (Zhang et al., 2018), adding to the diversity of EVs. These EV subtypes have a distinct mechanism of biogenesis and release that are coordinated through selected intracellular pathways. Studies on the heterogeneity of EVs are currently an ongoing topic with significant interest that is supported by new and more advanced isolation and characterization techniques.

The role of EVs in intercellular communication was first highlighted in the 1980s and since then, they have been shown to mediate physiological and pathological functions (Colombo et al., 2014; Raposo & Stoorvogel, 2013; Yáñez‐Mó et al., 2015). EVs consist of lipids, nucleic acids (DNA, mRNA, microRNA (miRNA/miR), and other non‐coding RNAs) and proteins (receptors, transcription factors, enzymes, extracellular matrix (ECM) proteins), that are distinct from the cells (Raposo & Stoorvogel, 2013). The proficiency of EVs to mediate intercellular communication relies on the systemic transport of these bioactive molecules to proximal and distal anatomical locations where they may induce signal transduction or mediate the horizontal transfer of information in specific recipient cells.

EVs represent functional components of the tumour cell secretome besides cytokines, growth factors, enzymes and glycoproteins. Recent developments in cancer biology have identified EVs as a part of the tumour secretome that are employed by tumours to establish local and distal microenvironments, complementing the free circulating tumour‐derived growth factors, extracellular proteins, and chemokines (Peinado et al., 2012). Emerging evidence indicates that tumour‐derived EVs carry functional molecular cargo and provide various autocrine and paracrine signalling cues that orchestrate: (1) initiation of metastasis through Epithelial‐Mesenchymal Transition (EMT); (2) synchronized preparation of pre‐metastatic niche via the recruitment and education of bone marrow‐derived cells (BMDCs), transdifferentiation of fibroblasts into myofibroblasts, vascular remodelling, coagulation and modulation of matrix biology; and (3) cancer immunoediting and subversion to evade immunosurveillance (Syn et al., 2016). EVs may be involved in an evolutionary mechanism adopted by cancer cells to ensure their survival and propagation at the site of metastasis.

While a plethora of papers have highlighted the many functions and pathways driven by EVs in cancer metastasis and progression, many of these studies have utilized isolated EVs derived from cultured cells that were then injected into animal models. These techniques provided valuable, initial insights into the role of EVs in cancer, however further research is needed to confirm the true biological function of EVs. In recent years, new approaches such as using genetically modified *Drosophila* and *C. elegans* have been developed to help understand the in vivo functions of EVs (reviewed in (Beer & Wehman, 2017)). Others have utilized zebrafish as a potent animal model to track circulating EVs in vivo with high spatiotemporal accuracy at the single EV scale (Hyenne et al., 2019). In parallel, new technologies such as intravital imaging have been developed to investigate the in vivo kinetics of systemically injected exogenous EVs in organs and target tumours (Lai et al., 2015; Takahashi et al., 2017; Zomer et al., 2015). Such new imaging strategies and modalities for monitoring the actual in vivo function of tumour‐derived EVs in metastasis will further elucidate the biological role of EVs in cancer.

## 2 EXTRACELLULAR VESICLES IN ORGANOTROPISM

Organotropic metastasis is the tendency of certain primary tumours to spawn and dictate secondary neoplasia at distant metastatic sites in specific organs. Distant metastasis involves multiple factors including infiltration and colonization of tumour cells, the kinetics (time to relapse/recur) of metastatic progression, and the host organ sites (Nguyen et al., 2009). The cellular origin of DTCs, genetic or epigenetic fluctuation of a DTC population, intrinsic properties of the tumour, tissue affinities and circulation patterns as well as systemic or local changes in the organ microenvironment have all been identified as critical determinants of distant organ infiltration and colonization (Nguyen et al., 2009).

The microenvironment at the secondary site is considered important in yielding organ‐specific metastatic cells with a full competence for aggressive colonization. The bone marrow, lung, brain, and liver parenchyma impose different selective pressures for the establishment of metastatic populations. These pressures arise from the distinct physiological barriers, unique composition of the microenvironment of each organ, as well as haematogenous dynamics such as circulation patterns and vascular wall accessibility. Under the selective pressure of the host microenvironment, DTCs acquire robust metastatic competence characteristics through genetic and epigenetic alterations (Nguyen et al., 2009). In addition to the genetic events, intrinsic abilities of the metastatic cancer cells to interact with the host microenvironment provides a selective advantage to malignant cells for adaptation in the secondary site (Obenauf & Massagué, 2015).

Recent evidence suggests that cancer cells adopt EV‐mediated regulatory mechanisms for organ‐specific conditioning that allows the redirection of metastasis. Specifically, tumour‐derived EVs prime the specific organ niches to facilitate metastasis even for tumour cells with poor colonization capacity for those sites. Evidence indicates that EVs bias the metastatic efficiency to different target organs due to their affinity for specific recipient cells, thus mediating non‐random patterns of dissemination (Hoshino et al., 2015; Syn et al., 2016). Hoshino et al. proposed that small EV‐derived integrins (ITGs) dictate organ‐specific targeting (Hoshino et al., 2015), thus determining organotropism to specific organs, in particular the lung, liver, and brain. Small EV‐derived ITGα6β4 and ITGα6β1 specify primary lung metastasis, while ITGαvβ5 directs metastasis to the liver and ITGβ3 to the brain. ITGα6β4 and ITGα6β1 promote tumour‐EV adhesion to lung‐resident fibroblasts and epithelial cells, thus governing lung tropism, while ITGαvβ5 promotes adhesion to Kupffer cells, mediating liver tropism. ITGs also interact with cell‐associated ECM molecules which may mediate EV uptake in specific target organs. Further, ITGs are also known to activate cellular signalling such as the proto‐oncogene tyrosine‐protein kinase Src (Hoshino et al., 2015). Activated Src has been shown to initiate pro‐migratory and pro‐inflammatory mechanisms via upregulation of the S100 gene in specific resident cells within distant tissue microenvironments (Hoshino et al., 2015). Similarly, another study has shown the role of ITGs in organotropism as demonstrated from the association of EV‐derived ITGα2β with brain metastasis, and that of ITGα4 with lymph node (LN) metastasis (Wan et al., 2018).

### 2.1 Extracellular vesicles in visceral metastasis

#### 2.1.1 Brain metastasis

Brain metastasis is more frequently observed in daily clinical practice, occurring in approximately 10%–30% of all cancer patients (Wrobel & Toborek, 2016). The progressive growth of metastasis in the brain tissue is usually detected in the advanced stage of the disease and most brain metastasis patients present multiple tumours at the time of diagnosis. The most frequent primary tumours metastasizing to the brain are lung cancer, breast cancer (BCa) and malignant melanoma (Eichler et al., 2011). To invade into the brain parenchyma, cancer cells have to pass through the blood‐brain barrier (BBB) (Arshad et al., 2010; Bos et al., 2010), which consists of the endothelium and surrounding cells (Abbott, 2013; Ballabh et al., 2004). The BBB is the major barrier for the passage of active molecules and circulating tumour cells from blood compartments to the brain, hence one of the key mechanisms of brain metastasis is the destruction of the BBB (Arvanitis et al., 2020).

Several studies have recognized the role of EV‐associated noncoding RNAs in increasing the endothelial cell permeability in the BBB and facilitating brain metastasis in BCa. Zhou et al. reported that EV‐associated miR‐105, derived from metastatic BCa cells, negatively regulates the tight junction protein zonula occludens 1 (ZO‐1), inducing endothelial cell permeability in the BBB and brain metastasis (Zhou et al., 2014). In another report, Tominaga et al. showed that EVs derived from metastatic BCa cells breach the BBB by targeting 3‐phosphoinositide‐dependent protein kinase‐1 (PDPK1), leading to the degradation of phosphorylated cofilin and severing of actin filaments via their cargo of miR‐181c‐5p (Tominaga et al., 2015). Once internalized by brain microvascular endothelial cells (BMECs), EVs derived from highly brain metastatic BCa cells have also been shown to destroy the BBB system and promote the invasion of metastatic cancer cells across the BBB, by transferring long noncoding RNA (lncRNA) GS1‐600G8.5 (Lu et al., 2020).

Several investigations have deciphered the nature of EV‐mediated interactions between metastatic tumour cells and brain microenvironment cells and delineated their prime role in the adaptation and progression of brain metastatic tumours. EVs, secreted by brain metastatic cells, have been determined to pre‐condition the brain microenvironment to enhance cancer cell outgrowth. In line with this, Rodrigues et al. have shown that brain metastatic cell‐derived EVs are enriched with cell migration‐inducing and hyaluronan‐binding protein (CEMIP), which promotes cancer cell colonization and brain metastasis by generating a pro‐metastatic environment. Mechanistically, uptake of CEMIP‐positive EVs by brain endothelial and microglial cells stimulates endothelial cell branching and the establishment of a pro‐inflammatory vascular niche by upregulating chemokines and cytokines known to promote brain vascular remodelling and metastasis (Rodrigues et al., 2019).

Cancer cells are well‐known to hijack several central nervous system (CNS) protective mechanisms and employ microglia and CNS border‐associated macrophages to achieve CNS colonization (You et al., 2019). Growing evidence points to the significant role of EV‐mediated communication between resident microglia, infiltrating macrophages and brain metastatic tumour cells that culminate in the colonization of the pre‐metastatic niche in the brain and ultimately, brain metastasis. In an effort to delineate the highly complex and selective process of brain metastasis in BCa, Xing et al. profiled lncRNAs in brain metastatic tumours and found that the loss of X‐inactive‐specific transcript (XIST) promotes brain metastasis in BCa. Mechanistically, loss of lncRNA XIST promotes metastatic progression of BCa by enhancing both stemness and aggressiveness of tumour cells through induction of EMT, activation of c‐MET via MSN‐mediated protein stabilization and the release of EVs containing miR‐503. They further demonstrated that EV‐associated miR‐503 promotes reprogramming of microglia from an M1 to an M2 phenotype by modulating signal transducer and activator of transcription 3 (STAT3) and nuclear factor kappa‐light‐chain‐enhancer of activated B cells (NF‐κB) pathways, followed by enhancing their programmed cell death ligand 1 (PD‐L1) expression that facilitates local immune suppression and tumour growth (Xing et al., 2018). Studies have also demonstrated the interaction of breast tumour‐derived EVs with Toll‐like Receptor (TLR) 2 on macrophages and the consequent activation of the NF‐κB signalling pathway in macrophages, resulting in the secretion of pro‐inflammatory cytokines such as interleukin (IL)‐6, tumour necrosis factor alpha (TNF‐α), granulocyte colony‐stimulating factor (G‐CSF), and C–C motif chemokine ligand 2 (CCL2; also known as monocyte chemoattractant protein 1, MCP‐1) (Chow et al., 2014). This TLR‐dependent stimulatory effect on macrophages was found to be, in part, mediated by multiple palmitoylated proteins located on the surface of cancer‐derived EVs (Chow et al., 2014). These studies demonstrate the immunomodulatory potential of EVs derived from metastatic brain tumours and their capacity to interact and induce a tumour‐propagating phenotype in resident microglial cells and infiltrating macrophages.

In the context of brain metastasis, extensive investigations have considered the crucial role of brain metastasis‐associated astrocytes. Compelling evidence indicates that astrocytes, the major glial cell in the CNS, play an intricate role in brain metastasis by establishing different modes of interactions with incoming primary and metastatic tumour cells (Wasilewski et al., 2017). Although our knowledge on the nature of this cross‐talk is still insufficient, recent seminal findings indicate EV‐based pro‐tumorigenic interactions between astrocytes and metastatic tumour cells. Astrocyte‐derived EVs have been shown to mediate an intercellular transfer of phosphatase and tensin homolog (PTEN)‐targeting miR‐19a to metastatic BCa cells, resulting in PTEN loss in brain metastatic tumour cells. This PTEN loss contributes to an increased secretion of CCL2 and the recruitment of IBA1+ myeloid cells that reciprocally enhance the outgrowth of brain metastatic tumour cells via enhanced proliferation and reduced apoptosis (Zhang et al., 2015). New insights gained from a series of in vitro and in vivo metastasis models have specified the importance of this tumour cell non‐autonomous PTEN downregulation by EV‐derived PTEN‐targeting miRNAs, particularly in BCa brain metastasis (Morad & Moses, 2019).

Deregulation of cellular bioenergetics is one of the emerging hallmarks of cancer (Hanahan & Weinberg, 2011). Reprogrammed glucose metabolism as a result of increased glucose uptake and the reliance of cancer cells on aerobic glycolysis even under normoxic conditions is one of the many strategies adopted by cancer cells to meet their biosynthetic demands associated with rapid growth and proliferation. In line with the novel metabolism‐centric regulatory role of tumour‐secreted EVs, Fong and co‐workers have shown that cancer cells adopt EV‐mediated mechanisms to suppress glucose uptake by non‐tumour cells in the pre‐metastatic niche and reallocate that glucose for their own utilization (Fong et al., 2015). Specifically, BCa‐derived EV‐associated miR‐122 was shown to modify glucose utilization by recipient astrocytes through downregulation of the glycolytic enzyme M2‐pyruvate kinase (PKM2) and glucose transporter 1 (GLUT1), leading to enhanced cancer cell proliferation, partially mediated by increased glucose availability to cancer cells (Fong et al., 2015). These results demonstrate that tumour‐derived EV‐associated miRNAs possess the capacity to reprogram systemic energy metabolism to facilitate circulating tumour cell colonization and expansion in the brain.

Given the well‐established role of EVs in matrix modulation, evidence indicates both a direct and indirect ECM‐modulatory role of EVs derived from brain metastatic tumours in the brain microenvironment. Maji et al. have shown that EVs derived from the MDA‐MB‐231 cell line are enriched with annexin A2 (AnxA2), which induces an increase in matrix metalloproteinase (MMP)‐9 expression in the brain stroma and contributes to extracellular proteolysis (Maji et al., 2017). AnxA2, as a tumour‐associated protein, has been implicated in many cancer‐associated functions, including plasminogen activation, actin‐cytoskeletal rearrangement (Rescher et al., 2008), cellular migration, adhesion and proliferation (Lokman et al., 2011; Maji et al., 2017). AnxA2 serves as a profibrinolytic co‐receptor for tissue plasminogen activator (t‐PA) and plasminogen and is involved in the generation of t‐PA‐dependent plasmin that is physiologically involved in fibrinolysis and neoangiogenesis (Hajjar & Acharya, 2000; Ling et al., 2004; Wang et al., 2011). Upon internalization by BCa cells, vesicular AnxA2 is hypothesized to form a complex with pro‐cathepsin B, t‐PA, plasminogen and AnxA2 light chain p11/S100A10, and expedite ECM degradation, plasmin generation and metastatic dissemination of the BCa cells to both lungs and brain (Maji et al., 2017). Besides modulating matrix biology, vesicular AnxA2 was found to promote angiogenesis via its association with endothelial t‐PA (Maji et al., 2017).

**FIGURE 1:**
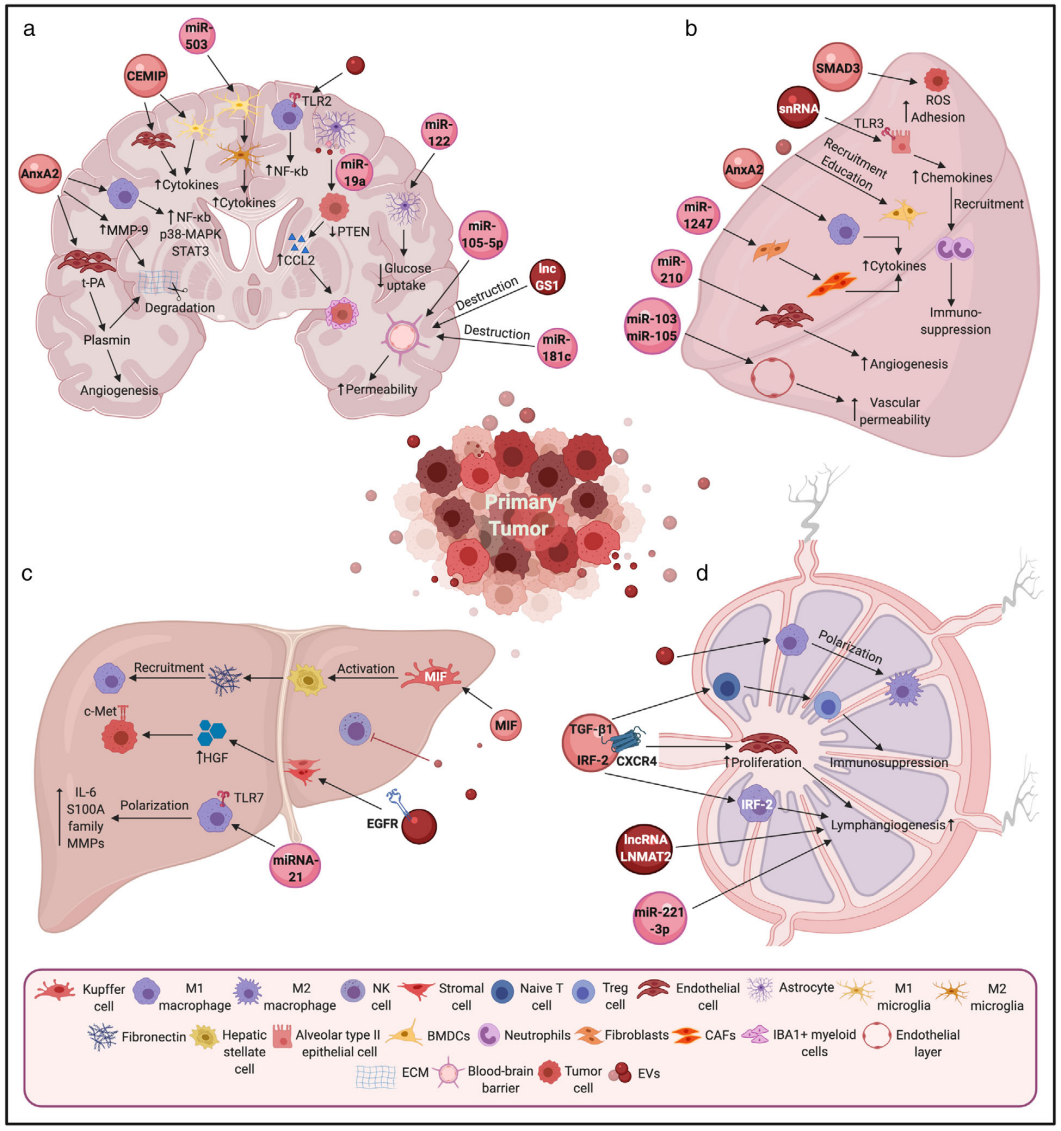
Decoding the role of extracellular vesicles in visceral metastasis. Several EV‐mediated pathways have been identified to drive metastasis in the brain (a), lungs (b), liver (c), and lymph node (d). The pre‐conditioning of secondary metastatic sites is a prerequisite for successful adaptation and colonization of engrafted tumour cells. EVs released by tumour cells have been proposed to travel to distant sites and help re‐organisation of the secondary site for successful tumour colonization via a number of pathways, including upregulation of pro‐inflammatory gene expression and immunosuppressive cytokine secretion, induction of phenotype‐specific differentiation, increased angiogenesis, vascular remodelling, modulation of matrix biology, deregulation of cellular bioenergetics and recruitment of specific stromal cell types

Xu et al. reported that human BMEC‐derived EVs stimulate an increased expression of S100A16 in recipient metastatic lung cancer cells. HBMEC‐derived EVs induce translocation of S100A16 from the cytoplasm to the nucleus in the recipient cells, which triggers the expression of prohibitin‐1, facilitating apoptosis‐resistance in metastatic lung cancer cells in the brain (Xu et al., 2019). Taken together, EVs play a crucial role in the symbiotic relationship between tumour cells and the brain microenvironment cells that supports adaptation and metastatic colonization in metastatic brain tumours (Figure 1a). In comparison to primary brain tumours, the EV‐based mechanisms of adaptation and progression in brain metastatic cancers are far less understood and predominantly focused on BCa brain metastasis. Establishing in vivo animal and relevant in vitro models that can provide a representative recapitulation of brain metastatic propensity of other cancers such as lung cancer and melanoma is essential to underscore the role of EVs in metastatic brain tumours and develop actionable anti‐metastatic therapeutic targets.

#### 2.1.2 Lung metastasis

Lung metastasis is frequently observed in a number of different cancers, including gastrointestinal tumours, renal carcinomas, BCa and malignant melanoma (Budczies et al., 2015). Pulmonary capillaries are lined with endothelial cells surrounded by a basement membrane with adjacent alveolar cells (Nguyen et al., 2009). To penetrate these structural barriers, metastatic cancer cells are proposed to express specific mediators that facilitate the relaxation of tight cell‐cell junction between endothelial cells and promote cancer cell invasion into the lungs (Gupta et al., 2007; Padua et al., 2008). After extravasation into the lung parenchyma, the majority of cancer cells perish via tumour‐stroma interaction, but a minority of these cells survive for anywhere from months to decades and establish metastatic tumours.

Previous studies have reported that EVs derived from metastatic cancer cells adopt three main strategies to establish metastasis in the lung; recruitment of immune cells, vascular remodelling and stromal alteration. Peinado et al. reported that metastatic melanoma cell‐derived EVs promote pre‐metastatic niche formation in the lung via recruitment and reprogramming of the BMDCs via transfer of activated c‐MET (Peinado et al., 2012). In another study, Liu et al. demonstrated the capacity of melanoma‐derived EVs, enriched in small nuclear RNAs (snRNAs), to activate TLR3 expression in alveolar type II epithelial cells for initiating neutrophil recruitment, immunosuppression and establishing a pre‐metastatic niche (Liu et al., 2016). Recently, Maji et al. revealed that Anxa2 carried by EVs, derived from metastatic BCa cells, activates the p38 MAPK, NF‐kB, and STAT3 pathways in macrophages, leading to the secretion of inflammatory cytokines and promoting the establishment of the pre‐metastatic niche in both brain and lungs (Maji et al., 2017).

In addition to promoting immune cell recruitment to the lungs, EVs can directly induce vascular remodelling. Feng et al. have shown that hepatocellular carcinoma (HCC) cells secrete EV‐packaged miR‐103 that can promote tumour motility by targeting endothelial junction proteins and increasing vascular permeability in vitro. Moreover, they showed that mice with xenografts of miR‐103‐expressing HCC cells exhibit higher levels of miR‐103 in EVs, higher vascular permeability and increased rates of lung metastasis (Fang et al., 2018).

Angiogenesis is also reported to contribute to lung metastasis. Kosaka et al. revealed that the neutral sphingomyelinase 2 (nSMase2) regulates secretion of miR‐210 contained in EVs, which affects the capacity for lung metastasis. In particular, they showed that EV transport of miR‐210 in endothelial cells promotes angiogenesis and enhances their metastatic initiation efficiency in lungs (Kosaka et al., 2013).

Stromal cell alteration induced by metastatic cancer cell‐secreted EVs plays an important role in establishing the pre‐metastatic niche in the target organ. Recently, Fang et al. have reported the communication between HCC‐derived EVs and fibroblasts in the metastatic niche. They revealed that HCCs secrete EVs containing miR‐1247‐3p, which directly targets B4GALT3, leading to the activation of β1‐integrin‐NF‐κB signalling in fibroblasts. These activated cancer‐associated fibroblasts (CAFs) subsequently induce the secretion of pro‐inflammatory cytokines, including IL‐6 and IL‐8, to boost lung metastasis (Fang et al., 2018). In addition, primary HCC cells have been shown to secrete EVs containing both SMAD Family Member 3 (SMAD3) protein and mRNA, which when delivered to recipient circulating HCC cells, regulate circulating tumour cell proliferation and adhesion and promote lung metastasis via the SMAD3‐reactive oxygen species (ROS) signalling pathway (Fu et al., 2018) (Figure 1b).

#### 2.1.3 Liver metastasis

The liver is the most common site of tumour metastasis and a major cause of death from gastrointestinal cancers, BCa and malignant melanoma (Hess et al., 2006). Intestinal mesenteric drainage leads into the hepatic portal venous system with the liver being the first port of call. Therefore, for gastrointestinal malignancies, the liver provides the best opportunity for cancer cells to extravasate and colonize within the hepatic parenchyma (Gupta et al., 2005). On the other hand, BCa or malignant melanoma do not have a similar chance of metastasizing to the liver, considering their anatomical location. Therefore, beyond the direct circulatory pathway, intercellular communication between metastatic cells and microenvironmental cells could play an important role in liver‐specific metastasis.

Compared to the vessels in the brain or the lung, the hepatic vasculature has a somewhat different structure. The hepatic vasculature lacks an organized sub‐endothelial basement membrane, therefore, metastatic cells can extravasate into the liver easier than into the brain or the lungs (Chambers et al., 2002). On the other hand, the liver parenchyma is rich in various kinds of cells, such as hepatocytes, hepatic stellate cells, sinusoidal endothelial cells, cholangiocytes, fibroblasts, dendritic cells, Kupffer cells, and lymphocytes. In addition, circulating and bone marrow‐derived immune cells can also be recruited to the liver. Therefore, the liver parenchyma is rich in immune cells and equipped with the innate immune system. These cells pose obstacles for cancer cells to develop metastasis in the liver (Brodt, 2016).

To date, several reports have indicated the potential of cancer cell‐derived EVs to aid the establishment of a pre‐metastatic niche in the liver. Costa‐Silva et al. have shown that pancreatic ductal adenocarcinoma (PDAC)‐derived EVs alter transforming growth factor (TGF)‐β production by Kupffer cells, leading to an increased fibronectin production by hepatic stellate cells and recruitment of bone marrow‐derived macrophages. They further identified macrophage migration inhibitory factor (MIF) as an essential trigger of the pre‐metastatic niche in the liver (Costa‐Silva et al., 2015). Pfeiler et al. found the additional role of pancreatic cancer cell‐derived EVs revealing they are not only internalized by resident Kupffer cells but also by the perivascular macrophages which support pre‐metastatic niche formation and subsequent liver metastasis (Pfeiler et al., 2019). miRNA‐containing EVs play an important role in liver metastasis. Colorectal cancer (CRC) cells secrete EVs enriched in miR‐21‐5p which polarizes macrophages through TLR7. These activated macrophages produce inflammatory cytokines such as IL‐6, promoting pre‐metastatic niche formation and liver metastasis (Shao et al., 2018).

In addition to activating pro‐inflammatory signalling processes to establish a pre‐metastatic niche, EVs modulate immune cell activity in liver metastasis. As previous reports have highlighted, the genetic depletion of natural killer (NK) cells or neutralization of pro‐apoptotic NK‐cell‐derived TNF‐related apoptosis‐inducing ligand (TRAIL) promotes liver metastasis (Milsom et al., 2013; Takeda et al., 2001). Several reports have shown that EVs from cancer cells bind to NK cells, resulting in tumour immune escape from NK surveillance (Ashiru et al., 2010; Lundholm et al., 2014). These results suggest a potential mechanism of EV‐mediated immune suppression in promoting liver metastasis.

On the other hand, stromal cell alteration can also contribute to the development of liver metastasis. Indeed, EVs derived from highly liver metastasis‐directed CRC cells recruit C‐X‐C chemokine receptor type 4 (CXCR4)‐expressing stromal cells, developing a metastatic‐designated microenvironment (Wang et al., 2015). In another study, epidermal growth factor receptor (EGFR) on EVs derived from gastric cancer (GC) cells can be transferred to the liver and internalized by liver stromal cells. The translocated EGFR suppresses miR‐26a/b expression in the liver stromal cells and activates the secretion of hepatocyte growth factor (HGF), which ultimately binds to the c‐MET receptor on the DTCs, promoting cancer proliferation in vitro and in vivo (Zhang et al., 2017) (Figure 1c).

#### 2.1.4 Lymph node metastasis

For many types of cancers, the process of metastasis involves lymphatic dissemination. Several articles have reported that the process of pre‐conditioning of sentinel lymph nodes (SLN) by tumour‐derived EVs is a prerequisite for the recruitment and growth of cancer cells. Hood et al. have shown that EVs derived from melanoma home to SLN and facilitate pre‐metastatic niche formation by increasing the expression of genes related to cell recruitment, ECM deposition and vascular proliferation in the LN (Hood et al., 2011).

To date, various kinds of molecules such as IncRNAs and proteins contained in EVs have been reported to promote lymphangiogenesis and contribute to LN metastasis. EVs secreted from cancer cells are mainly internalized by the lymphatic endothelial cells (LECs) and promote lymphangiogenesis. It has been shown that EVs, derived from cervical squamous cell carcinoma (CSCC), transfer miR‐221‐3p to LECs and promote lymphangiogenesis and lymphatic metastasis through downregulation of vasohibin1 (VASH1), an intrinsic factor with broad‐spectrum anti‐lymphangiogenic activity (Heishi et al., 2010; Zhou et al., 2019). Chen et al. have found that bladder cancer cells secrete EVs with a lncRNA LN metastasis‐associated transcript 2 (LNMAT2), that induces LEC tube formation and migration in vitro, and promotes tumour lymphangiogenesis and LN metastasis in vivo in a vascular endothelial growth factor C (VEGF‐C)‐independent manner (Chen et al., 2020). Once internalized by LECs, LNMAT2 epigenetically upregulates the expression of prospero homeobox 1 (PROX1) by recruiting heterogeneous nuclear ribonucleoprotein A2/B1 (hnRNPA2B1) and increases the level of H3K4 trimethylation in the PROX1 promoter, ultimately resulting in lymphangiogenesis and lymphatic metastasis. In addition, Li et al. found that EVs secreted by HCC cells increase the LEC proliferative rate and lymphatic tube formation ability via the guidance molecules such as CXCR4 (Li et al., 2018).

Furthermore, the effect of EVs on macrophages in LN and lymph metastasis have also been reported. In in vivo models, triple‐negative BCa cell‐derived EVs have been demonstrated to promote primary tumour growth and axillary LN metastasis by stimulating macrophage polarization to type 2 tumour‐supporting macrophages (Piao et al., 2018). Another study has reported that the delivery of interferon regulatory factor 2 (IRF‐2) by CRC‐derived EVs, to macrophages in SLN promotes VEGF‐C secretion, tumour‐associated lymphangiogenesis, and lymphatic metastasis. Macrophage ablation by clodrosome inhibits the effect of EVs on lymphatic remodelling and SLN metastasis, suggesting that macrophages are important targets of CRC‐derived EVs (Sun et al., 2019).

EVs have also been implicated in sabotaging anti‐tumour immunity in the lymphatic environment by recruiting immune‐suppressive regulatory T (Treg) cells. EVs derived from GC patients have been found to induce the differentiation of CD25^+^/CTLA4^+^/FOXP3^+^ Treg cells from naïve T cells through the effect of TGF‐β1 (Yen et al., 2017). Moreover, the TGF‐β1 found in EVs in GC patients has been associated with pathological stages and LN metastasis, thus, signifying the importance of EV‐associated TGF‐β1 as a predictive parameter for LN metastasis in GC patients (Yen et al., 2017) (Figure 1d).

### 2.2 Extracellular vesicles in bone metastasis

**FIGURE 2:**
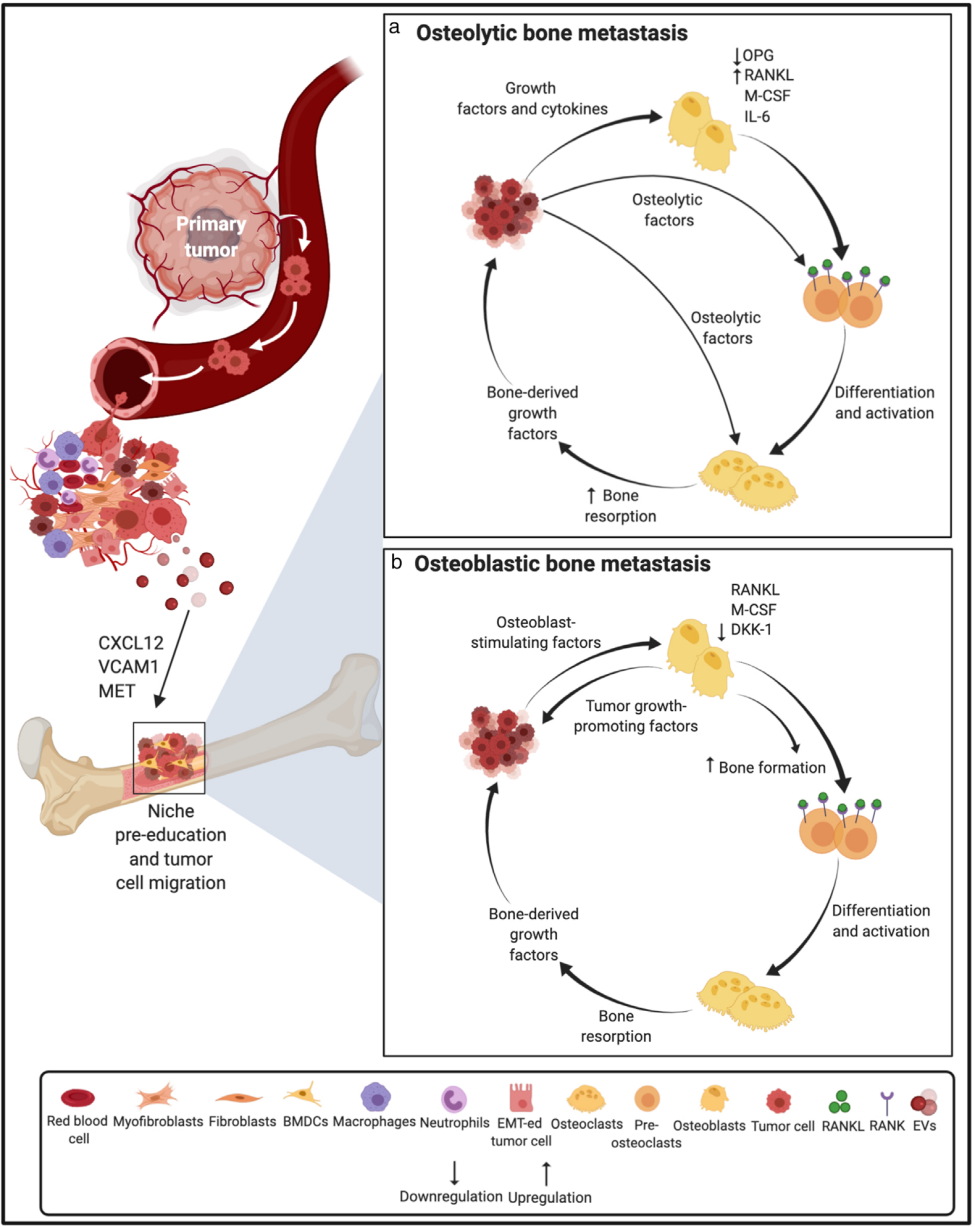
Schematic representation of the role of extracellular vesicles and tumour‐secreted factors in establishing pre‐metastatic niche and osteolytic/osteoblastic metastatic outgrowth in the bone. Primary tumour‐derived molecular components including tumour‐derived secreted factors and EVs play important roles in the modulation of the bone microenvironment, thus promoting metastasis. Prior to the arrival of tumour cells in the bone, EVs can facilitate the establishment of a pre‐metastatic niche in the bone via mechanisms such as the transfer of MET to BMDCs. (a) On establishing a foothold in the bone marrow niche, the osteolytic cancer cells (e.g. from breast cancer) secrete growth factors and cytokines that act on osteoclasts and osteoblasts in the bone microenvironment, such as PTHrP, VEGF, IL‐1, IL‐6, PGE2, TNF‐α, ET‐1 and BMPs. These factors increase the production of M‐CSF and RANKL, an osteoclast differentiating factor, while decreasing OPG (an osteoclastogenesis inhibitory factor) secretion from osteoblasts. The up‐regulated RANKL binds to its receptor RANK on the pre‐osteoclast surface and promotes the maturation of osteoclast precursors into functional osteoclasts and thus, osteolytic activation. Tumour cells also secrete osteolytic factors, most of which act via osteoblast RANKL, that further stimulate osteoclastic bone resorption. Bone resorption causes the release of PDGFs, BMPs, TGF‐β, IGF‐1 and calcium ions that in turn promote cancer cell proliferation, enable continued expression of osteoclast initiating factors and eventually perpetuate a cycle of osteolytic macrometastatic outgrowth. (b) Osteoblastic cancer cells (e.g. from prostate cancer) that have migrated to the bone, adapt to and modify the surrounding microenvironment by secreting osteoblast‐promoting molecules which increase osteoblast differentiation and proliferation including uPA, TGF‐β, VEGF, BMPs, TNF‐α, IGF‐1, Wnt1, WNT3A, ET‐1, PTHrP and adrenomedullin. Activation of the Wnt pathway and the decreased expression of the Wnt antagonist DKK‐1 stimulates osteoblast activity. In turn, enhanced osteoblast activity drives tumour progression by releasing IGF‐1, IL‐6 and IL‐8. Osteoclast activity is also activated in a predominantly osteoblastic lesion through osteoblast‐mediated osteoclastogenesis governed by the increased osteoblastic expression of RANKL and M‐CSF. Accelerated bone matrix degradation promotes the release of growth factors that further enrich the local milieu. Abbreviations: VCAM1, Vascular Cell Adhesion Molecule 1; CXCL12, CXC chemokine ligand 12; PTHrP, Parathyroid hormone‐related protein; PGE2, Prostaglandin E2; ET‐1, Endothelin‐1; BMPs, Bone Morphogenetic Proteins; M‐CSF, Macrophage colony‐stimulating factor; OPG, Osteoprotegerin; PDGF, Platelet‐Derived Growth Factor; IGF‐1, Insulin‐like growth factor 1; uPA, urokinase‐type Plasminogen Activator; and Wnt1, Wingless‐type MMTV integration site 1

Bone is the third most frequent and preferred site of metastasis, second to lung and liver (Coleman, 2001), and typically foretells a poor short‐term prognosis in cancer patients. Bone is a common site of tumour metastasis for BCa, prostate cancer (PCa), thyroid, lung, bladder, renal cancer and multiple myeloma (MM) (Macedo et al., 2017). Cross‐talk of DTCs with bone cells and the bone matrix results in the perturbation of homeostatic bone remodelling process and the formation of osteolytic (bone degrading) and/or osteoblastic (also known as sclerotic or osseous; bone‐forming) lesions. In recent years, accumulating evidence has unravelled the regulatory role of EVs in bone metastasis (Figure 2).

Recent findings from Nielsen and colleagues have shown that cell diffusion to distant skeletal sites in MM is favoured by EV‐associated procoagulant phospholipid and tissue factor (TF) activity (Nielsen et al., 2019). It has been determined that MM‐derived EVs inhibit osteogenesis in bone marrow‐derived mesenchymal stem cells (BMSCs) and that the elevated expression of miR‐103a‐3p may be involved in this osteo‐inhibitory effect in MSCs (Zhang et al., 2019). In the 5TGM1 murine model, MM‐derived EVs block osteoblast differentiation and functionality through the transfer of Dikkopf‐1 (DKK‐1) and reduction of runt‐related transcription factor 2 (Runx2), osterix (Osx) and collagen 1A1 (Col1A1) in osteoblasts (Faict et al., 2018). Furthermore, MM‐derived EVs can transfer bioactive lncRNA RUNX2‐AS1, arising from the antisense strand of Runx2, to MSCs and repress osteogenic differentiation, through a unique EV‐mediated lncRUNX2‐AS1/Runx2 pathway (Li et al., 2018). MM cell‐derived EVs also increase the expression of CXCR4, modulating pre‐osteoclast migration. Treatment of pre‐osteoclasts with MM cell‐derived EVs induces the expression of osteoclast markers such as cathepsin K (CTSK), MMP‐9 and tartrate‐resistant acid phosphatase (TRAcP), causing their differentiation into giant multinuclear osteoclasts with a capacity to excavate resorption lacunae (Raimondi et al., 2015).

Under hypoxic conditions, MM cells secrete IL‐32‐positive EVs which induce the nuclear translocation of NF‐kB, leading to the stimulation of osteoclastic differentiation and activation (Zahoor et al., 2017). The ability of MM‐derived EVs enriched with the EGFR ligand Amphiregulin (AREG) to block osteogenic differentiation of MSCs has been identified, through the release of the pro‐osteoclastogenic cytokine IL‐8 and activation of the EGFR pathway (Raimondo et al., 2019). Several onco‐miRNAs have been identified in MM such as miR‐21, ‐221/222, ‐125a, and ‐17/92 cluster (Pitari et al., 2015). Specifically, aberrant overexpression of miR‐21 is found in BMSCs adherent to MM cells. miR‐21 has been shown to suppress protein inhibitor of activated STAT3 (PIAS3), enhance the STAT3‐mediated receptor activator of nuclear factor‐kappa B ligand (RANKL) gene activation, and mediate RANKL‐induced osteoclastogenesis that ultimately contributes to the formation of bone lesions in MM (Pitari et al., 2015). EV‐associated RANK, the receptor for RANKL, from maturing osteoclasts can also accentuate RANKL reverse signalling in osteoblasts via Runx2, leading to bone formation (Ikebuchi et al., 2018).

It has been shown that non‐small cell lung cancer (NSCLC)‐derived EVs induce EGFR phosphorylation causing an increase of RANKL and osteoclastogenic markers (MMP‐9 and TRAcP) (Taverna et al., 2017). Specifically, EV‐associated AREG was found to be an important molecule in the induction of the EGFR pathway, inducing the typical phenotype of mature osteoclasts (Raimondi et al., 2015; Raimondo et al., 2019). Further, EV‐associated miR‐21 promotes osteoclastogenesis by targeting programmed cell death 4 (PDCD4), a known target of miR‐21 and a regulator of osteoclastogenesis (Xu et al., 2018).

Recent developments focused on re‐evaluating traditional aspects of the pathophysiology of PCa and BCa to bone metastasis have revealed the multifarious role of EVs in controlling bone homeostasis and cancer progression in the bone microenvironment (Patil & Soekmadji, 2021). Reports have shed light on the ability of EVs, derived from metastatic BCa cells, to shuttle protein cargo allowing BCa cell dissemination towards specific metastatic sites (Gangoda et al., 2017). Membrane proteins including Ceruloplasmin and Metadherin in the metastatic EVs are proposed to facilitate the site‐specific dissemination of BCa cells (Gangoda et al., 2017). Growing evidence has demonstrated the ability of BCa‐derived EVs to influence the behaviour of resident cells in the bone microenvironment and drive bone osteotropism. BCa‐derived EVs release the cytosolic protein L‐plastin (LCP1), which together with peroxiredoxin‐4 (PRDX4), stimulate RANKL‐independent osteoclastogenesis and thus induce osteolysis.

Hashimoto and colleagues have shown that hsa‐miR‐940 shuttled by PCa‐derived EVs promotes the osteogenic differentiation of human MSCs by targeting ARHGAP1 and FAM134A (Hashimoto et al., 2018). Interestingly, implantation of miR‐940‐overexpressing MDA‐MB‐231 osteotropic BCa cells induced osteoblastic phenotype in osteolytic lesions, mediated by the vesicular transfer of miR‐940 from cancer to host stromal cells (Hashimoto et al., 2018). This validates the crucial role of EVs in the pre‐metastatic dysregulation of bone remodelling in metastatic BCa.

**FIGURE 3:**
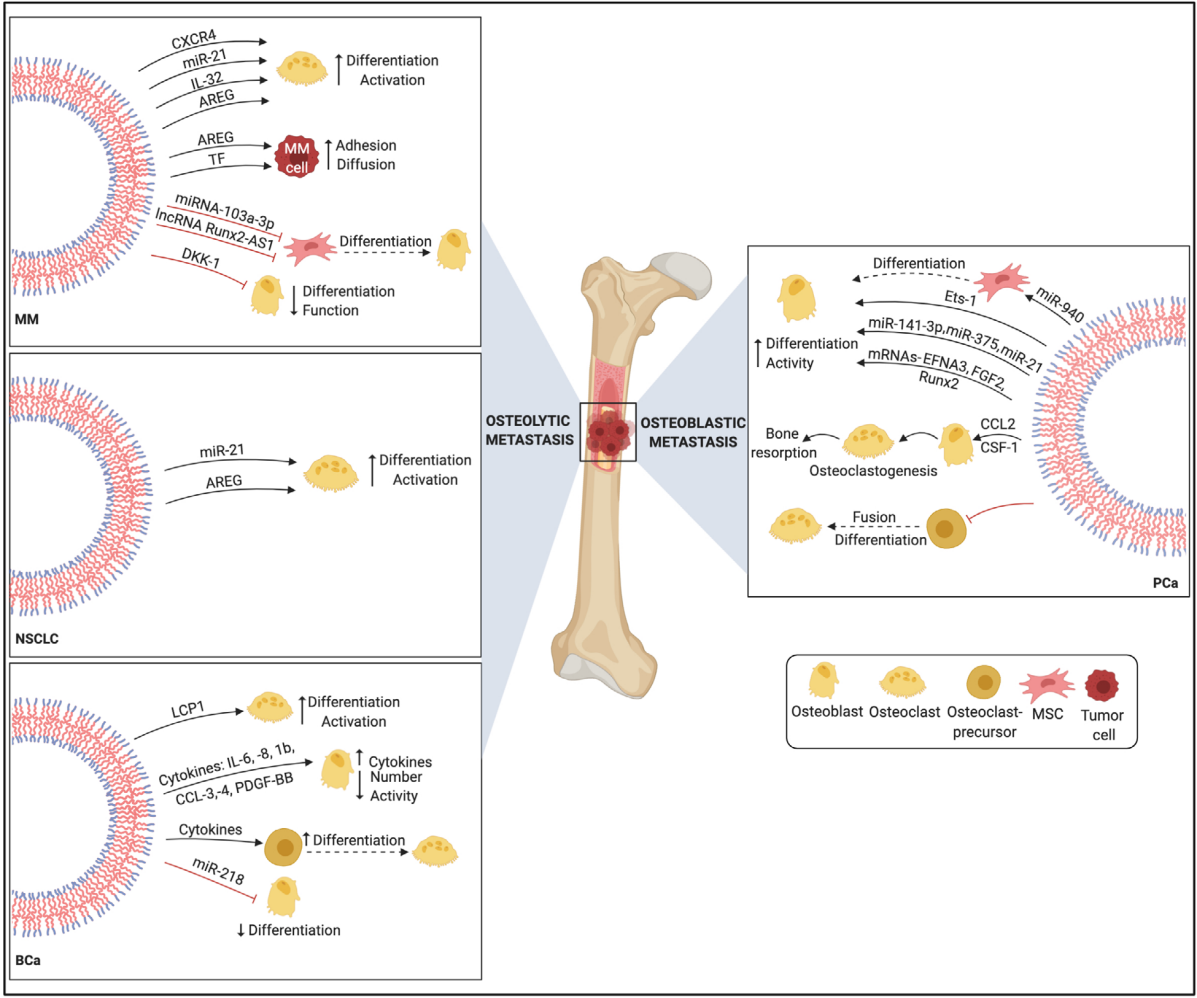
Mechanisms of extracellular vesicle‐mediated metastasis in skeletal metastatic cancers. In NSCLC, EVs carry pro‐osteoclastogenic miRNAs and EGFR ligands such as AREG that prompt NSCLC‐induced osteoclastogenesis and osteolytic bone invasion. Similarly, MM‐derived EVs enriched with AREG, lncRNA and onco‐miRs contribute to uncoupled bone remodelling and tumour‐induced osteolysis by blocking osteogenesis and stimulating osteoclast differentiation and function. Osteotropic BCa cells release EVs that condition the bone microenvironment toward an osteolytic phenotype via pro‐osteoclastogenic cargo and regulation of the osteoblast secretome. In PCa, EVs shuttle an array of osteoblast‐stimulating factors that facilitate osteoblast differentiation and activity and induce characteristic osteoblastic lesions in the bone metastatic microenvironment. Abbreviations: PDGF‐BB, Platelet‐Derived Growth Factor BB; FGF2, Fibroblast growth factor 2; EFNA3, Ephrin A3; CCL3, Chemokine (C‐C motif) ligand 3; and CCL4, Chemokine (C‐C motif) ligand 4

Another aspect worth considering is the role of EVs in promoting dormancy of BCa cells in the bone marrow. Co‐culture studies have shown that BMSC‐derived EVs induce dormant phenotypes in MDA‐MB‐231 cells through miR‐23b mediated suppression of a target gene, MARCKS, which encodes a protein that promotes cell cycling and motility (Ono et al., 2014). Conversely, BCa cells have been shown to prime MSCs to release EVs which in turn promotes dormancy in a subset of cancer cells and confers drug resistance, via their distinct miRNA cargo, such as miR‐222/223 (Bliss et al., 2016). All these findings illuminate the nature of the regulatory interactions, via EVs, between BCa cells and the bone environment in the evolution of tumour dormancy and bone metastasis (Figure 3).

## 3 CONCLUSIONS

As clinical oncology is progressing towards precision and personalized cancer medicine, a deeper understanding of the biology of metastasis has become increasingly acute. EVs are increasingly shown to be involved in the invasion‐metastasis cascade. EVs can facilitate organ‐specific metastasis and redirecting metastatic distribution which affects the organotropism in metastatic cancers. Identifying the organ‐specific molecular determinants/traits of EVs that drive organ‐specific patterns is an urgent need considering the dire clinical consequences associated with overt metastasis. Delineating the precise mechanisms adopted by EVs to promote the formation of pro‐metastatic environments at distant sites is crucial for understanding metastatic progression. Understanding EV‐driven mechanisms will help drive future potential therapeutic interventions to regulate EV function in metastasis which is an absolute requirement for gaining effective and elusive cure for patients with advanced metastatic cancer.

## CONFLICT OF INTEREST

The authors have declared no conflict of interest.
